# MLM-based typographical error correction of unstructured medical texts for named entity recognition

**DOI:** 10.1186/s12859-022-05035-9

**Published:** 2022-11-16

**Authors:** Eun Byul Lee, Go Eun Heo, Chang Min Choi, Min Song

**Affiliations:** 1 Department of Digital Analytics, Yonsei University, 50 Yonsei-ro Seodaemun-gu, 03722 Seoul, Republic of Korea; 2 Department of Library and Information Science, Yonsei University, 50 Yonsei-ro Seodaemun-gu, 03722 Seoul, Republic of Korea; 3 Department of Oncology, Asan Medical Center, University of Ulsan College of Medicine, Seoul, South Korea

**Keywords:** Bioinformatics, Named entity recognition, Language model, Artificial neural network

## Abstract

**Background:**

Unstructured text in medical records, such as Electronic Health Records, contain an enormous amount of valuable information for research; however, it is difficult to extract and structure important information because of frequent typographical errors. Therefore, improving the quality of data with errors for text analysis is an essential task. To date, few prior studies have been conducted addressing this. Here, we propose a new methodology for extracting important information from unstructured medical texts by overcoming the typographical problem in surgical pathology records related to lung cancer.

**Methods:**

We propose a typo correction model that considers context, based on the Masked Language Model, to solve the problem of typographical errors in real-world medical data. In addition, a word dictionary was used for the typo correction model based on PubMed abstracts. After refining the data through typo correction, fine tuning was performed on pre-trained BERT model. Next, deep learning-based Named Entity Recognition (NER) was performed. By solving the quality problem of medical data, we sought to improve the accuracy of information extraction in unstructured text data.

**Results:**

We compared the performance of the proposed typo correction model based on contextual information with an existing SymSpell model. We confirmed that our proposed model outperformed the existing model in a typographical correction task. The F1-score of the model improved by approximately 5% and 9% when compared with the model without contextual information in the NCBI-disease and surgical pathology record datasets, respectively. In addition, the F1-score of NER after typo correction increased by 2% in the NCBI-disease dataset. There was a significant performance difference of approximately 25% between the before and after typo correction in the Surgical pathology record dataset. This confirmed that typos influenced the information extraction of the unstructured text.

**Conclusion:**

We verified that typographical errors in unstructured text negatively affect the performance of natural language processing tasks. The proposed method of a typo correction model outperformed the existing SymSpell model. This study shows that the proposed model is robust and can be applied in real-world environments by focusing on the typos that cause difficulties in analyzing unstructured medical text.

## Background

Data digitization driven by the technological development of data collection and analysis methods has led to major changes in many fields and many text mining studies that extract and utilize meaningful information these data have been conducted [1–8]. In particular, Electronic Health Records (EHRs), which collect and manage patient health information in the form of electronic documents, are integrated with the hospital system and make it possible to utilize data in different medical fields [9]. EHRs contain a large amount of data and can be used as real-world evidence through appropriate analysis methods. However, the use of large-scale data of uncertain quality can lead to inaccurate or unreliable results [10].

Hersh et al. [11] and Zhou et al. [12] have shown that errors occur more frequently in text written quickly, such as in EHR (constitute approximately 5–17% of the total data). In the medical field, information accuracy is an important issue that is directly related to patient safety and efficient communication. For example, an error in a breast imaging report resulted in the exchange of incorrect information and affected a patient’s treatment [13]. Additionally, drugs with similar names can cause confusion and lead to erroneous drug prescriptions [14].

Typos appearing in these clinical data affect the performance of natural language processing (NLP) tasks in the medical field, such as part-of-speech (POS) tagging, information extraction, and information retrieval. Even a small number of typos have a negative effect on information retrieval tasks [15]. Especially in the task of extracting meaningful information from descriptively written unstructured text, such as Named Entity Recognition (NER), typo affects the extraction of important information from clinical data; therefore, handling typos is a necessary task before extracting information through NER [16]. However, few studies have been conducted to improve the quality of unstructured text data in the medical field [17].

Therefore, this study focused on the problems that hinder the accuracy of information and performance of text-based analysis in the medical field. Additionally, we identified how improvements in the quality of these data affect the performance of a natural language processing task for unstructured text. In particular, we evaluated our model and verified whether it was applicable to real data by extracting key diagnostic information from data in a real medical environment. Through this study, we sought to prevent medical accidents related to patient safety directly and increase the use of unstructured data by solving the problem of low-quality data from frequently represented typing errors.

The rest of the paper is organized as follows. We describe the proposed method in the Methods section. We evaluate the performance of typo correction with context and NER through data quality improvement in the "Experimental results" section. Finally, we conclude the research and suggest future work in the "Conclusions" section.

## Related work

### Typo correction in the medical field

The types of typos occurring in text are mainly divided into two types: ‘non-word typo errors’ and ‘context-sensitive typo errors.’ Non-word typo errors can be corrected in a simpler way than correcting context-sensitive typo errors. This study focuses on context-sensitive typo errors.

Correcting context-sensitive typo errors is a complex task because they require consideration of the relationship between the word to be corrected and the surrounding words. Context-sensitive typo errors can be classified into four types: homophone, typographical, grammatical, and spacing [18]. An example of each error is shown in Table 1. Studies using frequency-based, statistical-based, and deep learning-based methods have been conducted to solve context-sensitive typo errors. The frequency-based method works by assessing which word is most likely to appear in a sentence based on how often a word appears within that sentence. By contrast, the statistical-based method can correct typos based on context. Furthermore, deep learning-based typo correction studies can correct errors by considering the meaning of the context in a high dimensional embedding space.

**Table 1:** Types of errors and examples in context [18]

Error type	Cause of error	Example
Homophone error	Words that sound the same but are spelled differently	Peace/piece
Typographical error	Striking an incorrect key on a keyboard	From/form
Grammatical error	The user did not know exactly what the difference between grammars	Among/between
Spacing error	Wrong blank between words	Maybe/may be

Studies on typo correction in medical fields have been conducted for document accuracy. Senger et al. [19] have conducted a study to correct typos that occur when searching for drugs through electronic drug information systems in which information is digitized. By applying GNU Aspell, based on the Metaphone and Double Metaphone algorithms, candidate words were created and typos were corrected by ordering them based on the edit distance. The system that did not apply typo correction missed approximately 17.5% of the search results. By contrast, the system optimized with search auto-correction that applied a typo correction algorithm reduced noise when searching for drugs, thereby reducing the search time delay.

The National Library of Medicine (NLM) has a system to receive health questionnaires from users. Kilicoglu et al. [20] have applied a typo correction algorithm based on a phonetic algorithm and an edit distance algorithm to consumer health questionnaires collected from NLM. A group of candidate words for typo correction was generated based on a dictionary search, and word similarity, pronunciation similarity, and similarities calculating the number of matching words at the beginning and end of a word were used.

Workman et al. [21] have applied a typo correction algorithm to surgical pathology reports and emergency department progress and visit notes. To remove unnecessary words in the document, SPECIALIST Lexicon was used. This is a large-scale biomedical vocabulary corpus. Additionally, Word2Vec, a word embedding model, was used to embed the sentences. Typos were corrected by comparing the group of candidate words and typos by using the Levenshtein edit distance.

### Language models and named entity recognition

Language models can be divided into statistical-based methods and methods using artificial neural networks. The statistical-based language models find the word with the highest probability of appearing at a specific position based on previous words in the sentence through conditional probability. This probability is obtained from the corpus of a specific domain; therefore, it has a dependency on a specific domain. Moreover, there is the problem of long-term dependency as the length of the sentence increases. In addition, there is the problem of scarcity because a word that does not exist in the corpus has a probability of 0 even though it may be an appropriate word in that given sentence. In this case, the performance of the language model differs depending on the size of the corpus and how many different words the corpus contains.

Recently, transformer-based language models that use artificial neural networks [22], such as Bi-directional Encoder Representations from Transformers (BERT) and Generative Pre-trained Transformer (GPT), are showing the best performance. Since many texts are pre-trained in these models, they are domain-independent and can compensate for the scarcity problem that appears in existing language models. In addition, they are contextual language models that learn contextual information within a sentence.

The BERT process is divided into two tasks: pre-training and fine-tuning [23]. In the pre-training process, language modeling is conducted, and in the fine-tuning process, additional natural language is learned for the pre-trained model. Through this, BERT showed significantly better performance than the existing 11 natural language processing tasks.

Unlike existing word embedding models, such as Word2Vec [24], fastText [25], and GloVe [26], BERT embeds words by considering contextual information. In existing embedding models, each word has a fixed vector value. In BERT, information about homonyms (words that have the same spelling or pronunciation but have different meanings) cannot be considered depending on the context. BERT expresses a sentence as the sum of three embeddings: token, segment, and position. This reflects the contextual characteristics of a word. Token embedding uses the Word Piece embedding method to treat words that appear frequently in the document as a single word unit and divides words that rarely appear into sub-words. In an embedding model such as Word2Vec with a fixed vector, an out-of-vocabulary (OOV) problem occurs because a specific word does not exist in the word set. In the case of BERT, the OOV problem can be effectively dealt with by dividing rarely used words in the document into sub-words. Segment embedding divides sentences to predict the next sentence and position embedding embeds positional information from 1 to the maximum sequence length as a learned vector.

The Masked Language Model (MLM) is similar to the learning method of Word2Vec’s CBOW model by covering some words in a sentence and predicting the hidden words based on context information. $P\left({s}_{i}\right|{s}_{1},{s}_{2},\dots ,{s}_{i-1},\left[MASK\right],{s}_{i+1},\dots {s}_{t})$ is obtained when the $i$-th of a sentence $S$ consisting of $t$ words are masked. In general, previous language models learn unidirectional context and predict entire words, whereas MLM learns context information bidirectionally (from left to right and right to left) and predicts only the masked part of words. The [MASK] token for masking words is used during pre-training to learn contextual information in sentences while matching the correct answer to the masked part. These [MASK] tokens are not used during fine tuning, which creates a gap between the pre-training and fine-tuning processes. To reduce this gap, when randomly masking 15% of the total words, 80% of them are covered with [MASK] tokens, 10% are randomly replaced with other words, and 10% are kept as they are.

**Fig. 1:**
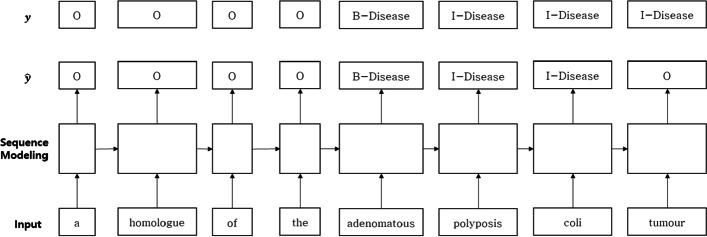
Example of named entity recognition. An example of sequence labeling to find most probable labeling of a sequence using BIO tagging for the entity extraction

NER is an NLP task that recognizes the named entity of a word or phrase with a specific meaning in the corpus. There are many types of entity according to different domains. For example, country, organization, and person are defined as entity names, which are extracted from within the corpus. NER is used for the preprocessing of major natural language processing tasks, such as chatbots and information retrieval. Unlike the general classification problem that outputs one value, it is a sequence labeling problem that receives an input sequence and outputs a sequence of the same length as the input sequence. The Begin, Inside, and Outside (BIO) format is used to recognize the object name. An example is shown in Fig. 1.

NER has been studied for text-based information extraction in various domains. In the medical fields, many studies have been conducted to recognize individual names, such as diseases, drugs, and DNA, and to extract information. An artificial neural network-based LSTM-CRF model has shown good performance, and recently, a pre-trained BERT-based model has shown the highest performance in NER.

Research with clinical NER has been applied from machine learning approaches to deep learning models. An active learning (AL) algorithm to minimize the annotation process has been proposed, which showed a performance of the F1-measure of 0.8 [27]. Recent NER studies based on clinical data have focused on neural word embeddings in the unlabeled clinical NER corpus [28], studies applying deep learning models [29], and transformer-based models [30] to extract clinical concepts and evaluate the performance of the clinical NER system.

## Methods

**Fig. 2:**
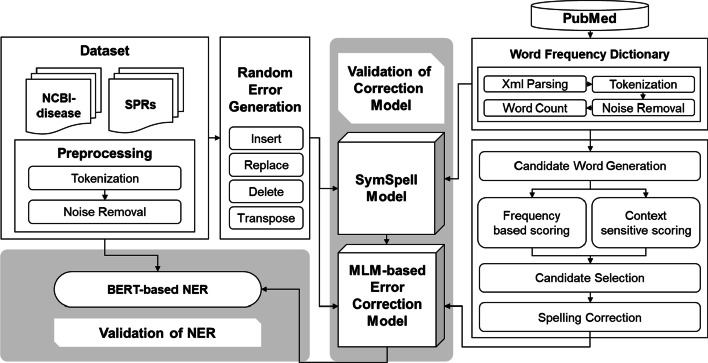
Research overflow. Two datasets, such as NCBI-disease and SPR, were used to extract important information from the unstructured data of a medical dataset. Two processes, such as spelling error correction and NER, were performed and verified to identify whether low-quality data with many typos affected the performance of information extraction

In this section, we describe the dataset, error generation, typo correction, MLM-based candidate word selection, and deep learning-based NER extraction. We examined whether low-quality data with many typos affected the performance of information extraction. To this end, two processes were performed to extract key information from low-quality data. First, we corrected typos in two datasets: NCBI-disease data and surgical pathology records (SPRs). Second, we identified NER after these data were refined through typo correction. Figure 2 illustrates the structure of the overall model. Detailed descriptions of each method are provided in the following subsections.

### Dataset and preprocessing

#### Dataset

To examine the effect of improving the quality of real-world medical data on the performance of the NLP task such as typo correction and NER, we used two datasets, NCBI-disease [31] and SPRs.

The NCBI-disease dataset [31] is used as a benchmarking dataset for NER in the medical domain. These data consist of 793 PubMed abstracts manually annotated with disease mentions and concepts from Medical Subject Headings (MeSH) or Online Mendelian Inheritance in Man (OMIM).

**Fig. 3:**
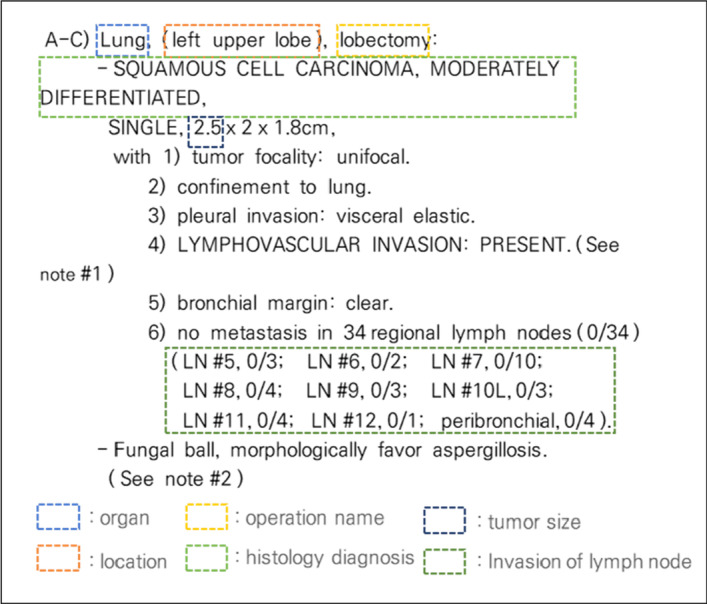
Example of the contents of the test result in the SPR dataset. The first part is the name of the organ, location, operation name, histology diagnosis, tumor size, and invasion of lymph node in order. We excluded invasion of lymph node from the range of information extraction for our work

The SPR dataset used to classify lung cancer staging. It was newly provided by the Asan Medical Center [32] with permission to conduct this study. Annotation was conducted for 3 months (from March 2020 to May 2020) by two medical experts from Asan Medical Center. After annotating a total of 47,180 data individually, 40,443 data with matching results were used as experimental data. The intra-annotator agreement was 85.72%. In total, 40,443 diagnosis results were composed of five columns, including the date and time of the prescription, test code, and text of the test result. We extracted the type of test, test institution, test location, result, and size of cancer from the text of the test result. Types of tests included Proliferating Cell Nuclear Antigen (PCNA), needle biopsy, bronchial washing, and pleural fluid. While the name of the test written in the text of the test result may be the same, there were some author-related differences in descriptions and terms. Figure 3 shows an example of the text of the test results, which is the input data of the model.

### Random error generation

There is no benchmark dataset for evaluating typo correction-related studies in the medical field; therefore, most studies have used a method of randomly generating and evaluating typos for evaluation. For example, Lai et al. [17] have analyzed the types of typos in medical text and classified them into insertions, deletions, substitutions, and transpositions, and conducted experiments based on these four types. In addition, Workman et al. [21] have categorized spelling error types of surgical pathology notes to insertion, omission, transposition, wrong letter, or mixed/multiple error types. Among them, the multiple/mixed error types appeared in a very small proportion. Lee et al. [18] have defined seven error types, such as omission of a letter, addition of a letter, single letter instead of double letter, double letter instead of single letter, substitution of one letter, interchange of two adjacent letters, and two or more of the same type or of different types. Based on these previous studies [16, 18, 21], we first defined four types of common typo that occurred frequently (Table 2).

**Table 2:** Type of Errors and Examples

Error type	Example
Insert	Randcom
Delete	randm
Replace	randcm
Transpose	randmo

Four representative error types suggested in previous studies on typo correction, such as insert, delete, replace, and transpose. Example typos of the word ‘random’. The ‘example’ column shows typos generated of each error type when the string (‘rand’, ‘om’) is randomly selected.

We split each word into two strings to create candidate words. For example, the word ‘random’ is made up of seven string combinations: [(‘’, ‘random’), (‘r’, ‘andom’), (‘ra’, ‘ndom’), (‘ran’, ‘dom’), (‘rand’, ‘om’), (‘rando’, ‘m’), (‘random’, ‘’)]. If the length of the string was less than one, such as (‘’, ‘random’), (‘random’, ‘’), they were excluded from the candidate group. One of the five remaining candidate groups was randomly selected to generate one of four error types. In the case of insert and replace, one of the alphabets A-Z, a-z was randomly selected and inserted or replaced between two strings. In the delete case, the first character of the right string was removed. The order of the first and second character of the right string was changed in the transpose case. Table 2 shows the typos generated according to the error generation procedure of each error type when the string (‘rand’, ‘om’) was selected.

Table 3 shows the sizes of training and test data in two datasets each. In SPRs, one line of the text content of the test result in excel file was defined as one sentence and used as the input of the model.

For NER experiments, the annotation of the SPR dataset was performed in two steps. First, the rules were composed and labeled by the researchers. Two experts in the lung cancer field from Asan Medical Hospital [32], who provided the dataset, cross-checked and performed secondary verification. Finally, cases where both experts were tagged with the same entity were set as named entities.

**Table 3:** Data specification

Datasets	Train/Test data	Sentences	Tokens
NCBI-disease	Train	6347	159,670
	Test	940	24,497
SPRs	Train	39,443	2,050,125
	Test	1000	49,668

Two datasets were divided into training and test data to evaluate the performance of typo correction and NER. The total number of sentences and tokens in the NCBI-disease dataset were 7287 and 184,167, respectively. The total number of sentences and tokens in SPR dataset were 40,443 and 2,099,793, respectively. The proportions of training data in each dataset were 87% and 98%, respectively.

#### Candidate word generation

In this study, the candidate group of words to be corrected was selected using the Edit distance algorithm. The typo was corrected by scoring each word candidate group in consideration of the frequency of words in the dictionary and the context within the sentence. The SymSpell algorithm was used to generate a candidate group of words to be modified [33]. In general, four types of text correction processes were performed to create a word candidate group based on the Edit distance: delete, transpose, replace, and insert. However, the SymSpell algorithm reduced computation amount by using only the delete approach. Therefore, we trained the model using PubMed abstracts (approximately 25.4GB of literature updated in December 2019) to optimize the SymSpell algorithm for the medical domain. In addition, word dictionary was created that summarized words and their frequency using the PubMed abstracts. If the frequency of any word was < 20 in the entire collection, then it was excluded from the word dictionary. As a result, 2,370,526 words were included in the dictionary. A group of word candidates was generated using the SymSpell algorithm based on this generated word dictionary.

### Spelling error correction

#### Masked language model-based candidate selection

We used a score that combines a frequency-based and context-based scores to find an appropriate word to correct any typo among the generated word candidates. The formula is as follows:

$$\left(FinalScore\right) = \lambda \left(FrequencyScore\right) + (1- \lambda )\left(ContextSensitiveScore\right)$$

Using the MLM method, scores were obtained in consideration of the context within the sentence. In particular, a BERT-based pre-trained MLM model was used. By adding a dense layer to the pre-trained model, we calculated the probability of a specific word in the masked part of the input sentence and used it as a score for the context. Table 4 shows the structure of the model added to the pre-trained BERT model.

**Table 4:** Structure of the candidate word selection model

Layer	Output shape
Input	(None, None, 768)
Dense	(768, 768)
Layer normalization	(768,)
Output	(None, None, 30,522)

We added a dense layer of (768, 768) to the pre-trained BERT model to consider context of text using MLM. Input layer is (None, None, 768), Output layer is (None, None, 30,552), and Layer Normalization is (768,) to modify inputs for the next layer.

**Fig. 4:**
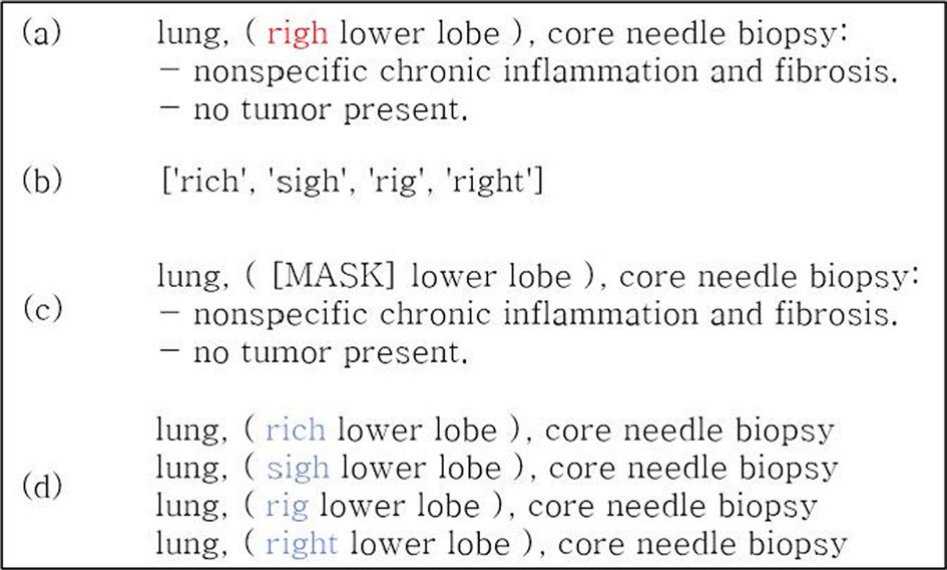
Example of the candidate word selection process. The word ‘righ’ in (**a**) should be corrected to ‘right.’ (**b**) shows the generation of candidate word to correct typos through the optimized SymSpell algorithm. In (**c**) and (**d**), the part where the typo appears is masked and replaced with a candidate word. The probability of the word entering a specific position in the sentence is calculated through the MLM model

Figure 4 shows an example of the process of finding an appropriate cored word from the generated candidate word group from our SPR dataset.

### Deep learning-based named entity recognition extraction

We performed fine-tuning by adding a dense layer to the pre-trained BERT to extract key information from medical data. The hyper-parameters used for fine tuning are shown in Table 5. The output value ${y}_{i}$ of the model equals the probability that the input value ${x}_{i}$ belongs to n tags of each dataset. It has a vector of (1, n). The softmax function was used to learn the probability that the model belongs to $\text{j}$ tags. When ${a}_{k}$ is the probability value of the $\text{k}$th tag among n tags, the probability that the $\text{k}$th tag is correct is ${y}_{k}$. The formula of softmax function is as follows:

$${y}_{k}=\text{e}\text{x}\text{p}\left({a}_{k}\right)/\sum _{i=1}^{n}(\text{e}\text{x}\text{p}({a}_{i}))$$

As a result of the model, each word was tagged as one of [B-Disease, I-Disease, O] for the NCBI-disease dataset, and [B-ORGAN, I-ORGAN, B-LOCATION, I-LOCATION, B-OPNAME, I-OPNAME, B-HISTOLOGIC DIAGNOSIS, I-HISTOLOGIC DIAGNOSIS, B-TUMOR_SIZE] for the SPR dataset. Through this, it was possible to extract the key information from the SPRs, such as organ, location, operation name, histology diagnosis, and tumor size.

**Table 5:** hyper parameters

Hyperparameter	Value
Learning rate	3e-5
Epochs	3
Max sequence length	178
Batch size	16
Optimizer	Adam
Activation function	Softmax

Information of fine-tuning process using pre-trained BERT, such as learning rate, epochs, max sequence length, batch size, optimizer, and activation function

## Experimental results

### Validation of context-based typo correction

We evaluated the SymSpell algorithm optimized for medical data and the context-based typo correction model with the NCBI-disease and SPR datasets. We compared the performance of the SymSpell algorithm and typo correction models when contextual information was included. Since a word with a typo among all words appeared with a very low probability compared with a word without a typo; therefore, the number of values corresponding to each class was unbalanced. Hence, an F1-score was used for quantitative performance evaluation.

Typos were randomly generated approximately in 16% and 7% of words in the NCBI-disease and SPR datasets, respectively. The number of tokens in each dataset differed more than twice; therefore, the error ratio was adjusted to match the number of four error types. Table 6 shows the number of typos of the four error types created in the NCBI-disease and the SPR datasets. The performance of the typo correction model was assessed in the generated sentences containing typos.

**Table 6:** Error type and number per dataset

Datasets	Type of typo				No Typo	Total
	Replace	Delete	Transpose	Insert		
NCBI-disease	1014	1073	911	946	20,553	24,497
SPRs	965	948	877	827	46,051	49,668

The total number of tokens was 24,497 in the NCBI-disease dataset. the total number of tokens with four error types was 3944, accounting for 16.10% of words. The total number of tokens without error (No typo) was 20,553, accounting for 83.91% of words. The total number of tokens was 49,668 in the SPR dataset. The total number tokens with four error types was 3617, accounting for 7.28% of words. The total number of tokens without error was 46,051, accounting for 92.72% words.

Table 7 shows the typo correction performance for each dataset. Assessment of the typo correction model was performed in the generated sentences containing typos. In the two datasets, the F1-scores marked in bold were 0.72 and 0.73, which improved performance approximately 5% and 9% in the model considering contextual information when compared with cases where contextual information was not included.

**Table 7:** Typo correction performance in the NCBI-disease and SPR datasets

Datasets	Algorithms	Precision	Recall	f1-score	Support
NCBI-disease	SymSpell	0.62	0.7	0.67	3944
	Proposed model	0.65	0.81	**0.72**	
SPR	SymSpell	0.59	0.7	0.64	3671
	Proposed model	0.70	0.76	**0.73**	

The occurrences of each type of word. ‘support’ means the number of words in the data. The performance of the proposed model was improved by 5% and 9% for each dataset, respectively.

**Fig. 5:**
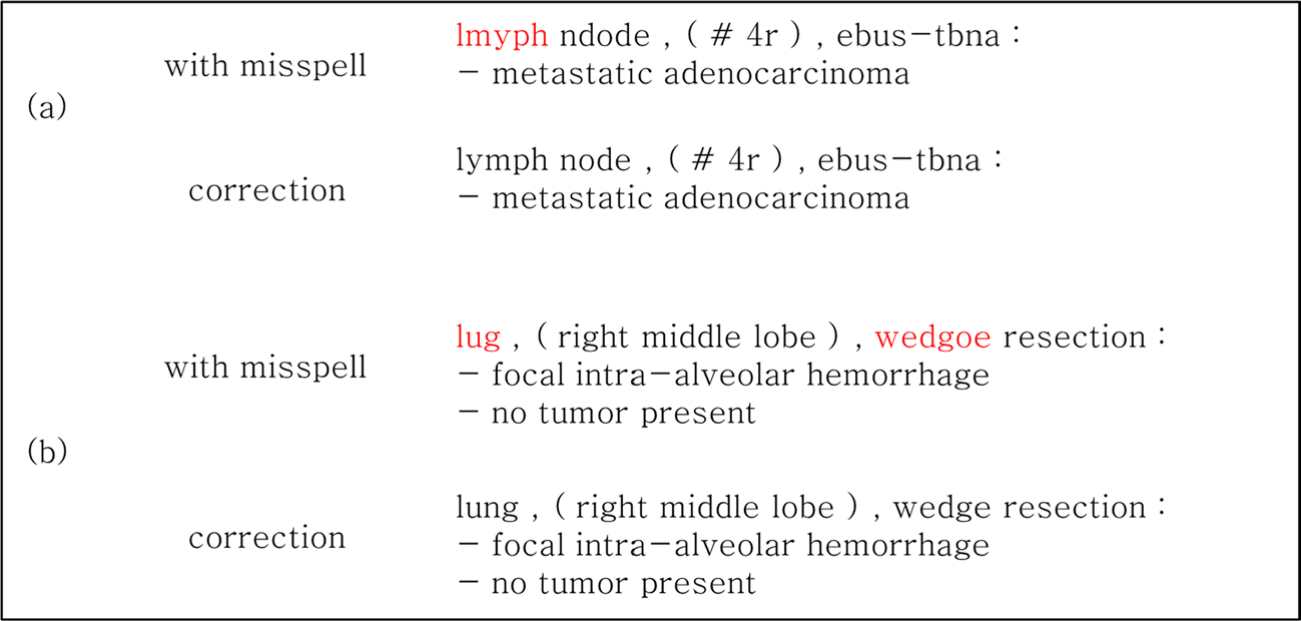
Example of typo correction through each model. In the case of (**a**), ‘lmyph’ is originally ‘lymph,’ which was corrected through the model. In the case of (**b**), ‘lug’ and ‘wedgoe’ mean ‘lung’ and ‘wedge,’ which were the body organs and test names, respectively. Both words were corrected

Figure 5 shows an example of correcting typos in the SPR dataset. If there was a typo in the part containing important information, it was confirmed that the word was corrected through the model.

### Validation of named object recognition through data quality enhancement

**Fig. 6:**
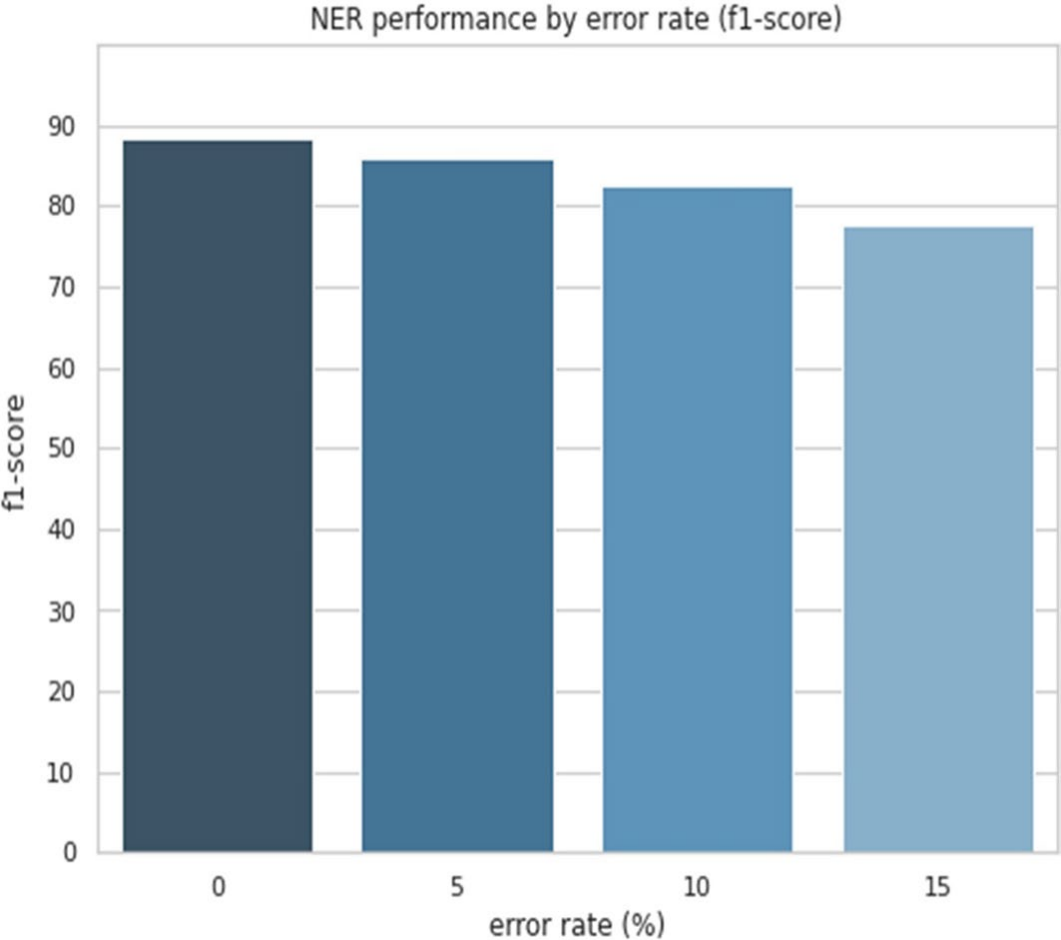
NER performance by error rate in the NCBI-disease dataset. The x-axis represents the error rate (%) and the y-axis represents the F1-score in NCBI-disease dataset. As the error rate increases, the F1-score tends to decrease

We showed the effect of typos on the performance of NER by randomly generating 5–15% of typos in the NCBI-disease dataset. In well-refined data without typos, the F1-score was 0.89. Interestingly, the F1-score was reduced to 0.85 when 5% of typos were included, 0.82 when 10% were included, and 0.77 when 15% were included (Fig. 6).

When 16% of typos were included in the NCBI-disease dataset, the average F1-score ﻿for NER marked in bold was 0.77. After the typo correction model was applied, the performance was improved by approximately 2% (F1 = 0.79). The detailed performance evaluation results in each case are shown in Table 8.

**Table 8:** NER performance before and after typo correction in the NCBI-disease dataset

NER	Precision		Recall		f1-score		Support
	Typos	Typo correction	Typos	Typo correction	Typos	Typo correction	
B-Disease	0.96	0.95	0.73	0.74	0.83	0.84	960
I-Disease	0.97	0.97	0.66	0.71	0.79	0.82	1087
	Typos			Typo correction			
Accuracy	0.70			0.72			2047
f1-score	**0.77**			**0.79**			

Dataset included 16% errors. The two tagged values, B-Disease and I-Disease, appeared 960 and 1087 times, respectively.

In the SPR dataset, the performance of NER was evaluated when 7% of typos were included. As shown in Table 9, the average F1-score marked in bold was 0.6 when typos were included. The NER performance increased to 0.85 after correcting the typos (15% improvement). This confirmed that typos had a significant effect on extracting information from the unstructured text.

**Table 9:** NER performance before and after typo correction in the SPR dataset

NER	Precision		Recall		f1-score		Support
	Typos	Typo correction	Typos	Typo correction	Typos	Typo correction	
B-ORGAN	0.97	1.00	0.31	0.82	0.47	0.90	1022
I-ORGAN	1.00	1.00	0.37	0.73	0.54	0.84	196
B-LOCATION	1.00	1.00	0.25	0.79	0.41	0.88	625
I-LOCATION	1.00	1.00	0.26	0.81	0.41	0.89	1338
B-OPNAME	0.90	0.98	0.54	0.97	0.68	0.97	822
I-OPNAME	1.00	1.00	0.46	0.96	0.63	0.98	803
B-HISTOLOGIC DIAGNOSIS	1.00	1.00	0.83	0.99	0.91	0.99	70
I-HISTOLOGIC DIAGNOSIS	1.00	1.00	0.90	1.00	0.95	1.00	114
B-TUMOR_SIZE	1.00	1.00	0.99	0.99	1.00	1.00	222
	Typos			Typo correction			
Accuracy	0.40			0.87			2047
f1-score	**0.60**			**0.85**			

Dataset included 7% typos. There are total seven tag values. Among them, four entity types, such as ORGAN, LOCATION, OPNAME, and HISTOLOGIC DIAGNOSIS, were located both in B and I, respectively. TUMOR_SIZE was located only in the B format.

## Discussion

In this study, we focused on the problems that deteriorate the accuracy of entity extraction and the performance of text-based analysis in the medical field. Typos frequently appear in unstructured texts and have a negative effect on the performance of text-based information extraction. Therefore, we sought to improve the performance of extracting important information from unstructured medical texts by resolving the typo problem in unrefined text. We verified that typos occurring in text have a negative effect on the performance of natural language processing tasks. Moreover, we showed an improvement in NER performance after typo correction.

Interestingly, there was a difference in NER improvement between the NCBI-disease and SPR datasets. The F1-score of NER detection increased by 25% in the SPR dataset following the typo correction model, whereas detection in the NCBI-disease dataset increased only by 2%. The NER is a sequence labeling process; therefore, recognition is affected according to the preceding and following words. This means that recognition of long entity names will be less affected by typos when compared with short entity names.

**Fig. 7:**
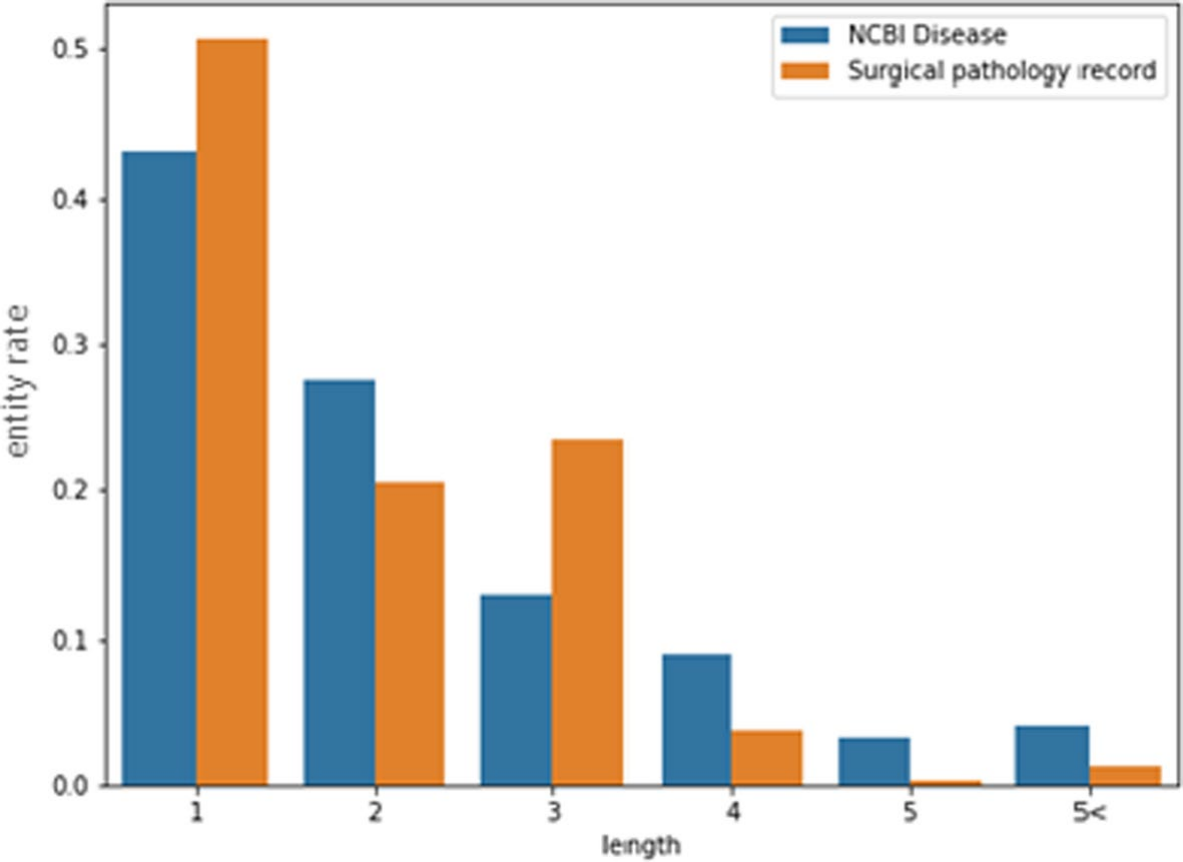
Comparison of entity ratio by entity length in NCBI-disease and SPR datasets. The x-axis represents the entity length, and the y-axis represents the proportion. The blue and orange bars represent the NCBI-disease and SPR dataset, respectively

Figure 7 compares the entity proportion by length in the two datasets. The ratio of a single word entity occupied half of the total entities in the SPR dataset; more than 90% of entities had a maximum length of 3. By contrast, entities with a length of 4 or more occupied approximately 20% of the words in the NCBI-disease dataset. This may be why the NCBI-disease dataset showed little difference in performance before and after typo correction. In addition, these characteristics differed depending on the entity name existing in the SPR dataset. For example, there are five entity names in SPR dataset, such as ORGAN, LOCATION, OPNAME, HISTOLOGIC DIAGNOSIS, and TUMOR_SIZE. Among them, HISTOLOGIC DIAGNOSIS is the longest entity with an average length 6. The F1-score of this entity name increased by 8% for Begin and 5% for Inside after correcting for typos. Hence, this entity was less affected by typos than the other, shorter entities.

We confirmed that the typographical correction model proposed in this study helps extract accurate information from unstructured data. We also reviewed that some exceptional cases that may occur infrequently in our real-world dataset. For example, in cases reporting the size of cancer, the contents written as ‘2.1 × 1.3 × 1 cm’, the largest value ‘2.1’ should be extracted. In most cases, this entity was written in the form of ‘Number × Number × Number;’ however, sometimes it was written in a different way, for example as ‘1.2 cm IN GREATEST DIMENSION.’ Although this case occupies a small proportion of the total data, a rule must be created that considers the number of all cases to apply a rule-based model. This task is labor intensive and inefficient because when a new exception appears, it is not possible to extract information from the existing rules. Therefore, rules ought to be modified again. We supplemented the limitation of rule-based information extraction by applying the BERT-based NER model to unstructured text in real-world medical fields. In this way, the proposed typo correction model can contribute to accurate information extraction from unstructured data.

Studies of pre-trained BERT were conducted based on data from various domains. There are representative models, such as BioBERT [34] and ClinicalBERT [35], for the medical domain. In particular, Med-BERT [36] is pre-trained on more than 2,800 structured EHR dataset. The BERT-base model used in this study was trained with Wikipedia and BookCorpus, not domain dependent data; therefore, additional verification is needed to determine its suitability for the medical domain. A future study will select an appropriate pre-trained model by comparing the performance of BioBERT [34], ClinicalBERT [35], and Med-BERT [36] when correcting typos in real-world medical data. In addition, it is expected that the performance of typo correction will be improved by constructing a word dictionary based on a corpus related to clinical data, such as the MIMIC-III Clinical Database rather than the scientific literature PubMed abstracts.

## Conclusion

Data digitization has led to vast amounts of patient data being accumulated daily, such as EHRs in real-world medical fields. Together with changes in technology, there is an active movement to build and analyze accumulated data platforms in medical fields. In particular, a large proportion of EHR data is written descriptively without a standard pattern. Extracting information, for example to judge a patient’s lung cancer staging from unstructured text, is necessary in real-world medical environments. However, the use of delimiters or abbreviations in text differ depending on the record writer. This necessitates the application of a robust methodology to resolve these exceptions. In addition, it is difficult to extract and structure important information because of frequent typos.

The purpose of this study was to propose a robust model that extracts the necessary information, even in undefined exceptions and typo situations, that can be applied to real-world medical fields. To this end, two tasks were performed: correcting typos in the data and extracting information by utilizing NER in the corrected text. Taken together, the results showed an effective model that can be applied to real world environment by focusing on problems that cause difficulties in analysis. In addition, our experiments showed that typos occurring in text data have a negative effect on the performance of natural language processing tasks.

## Data Availability

The data that support the findings of this study are available from the Asan Medical Center; however, restrictions apply to the availability of these data. In this study, data were used under license and are not publicly available. Data are available from the authors upon reasonable request and with permission of Asan Medical Center. The NCBI-disease data are available at https://www.ncbi.nlm.nih.gov/CBBresearch/Dogan/DISEASE/.
